# Supplementary figures and images for: Middle ear effusion, ventilation tubes and neurological development in childhood
Source: PLoS One. 2023 Jan 13;18(1):e0280199. doi: 10.1371/journal.pone.0280199 (PMC9838841; doi:10.1371/journal.pone.0280199)

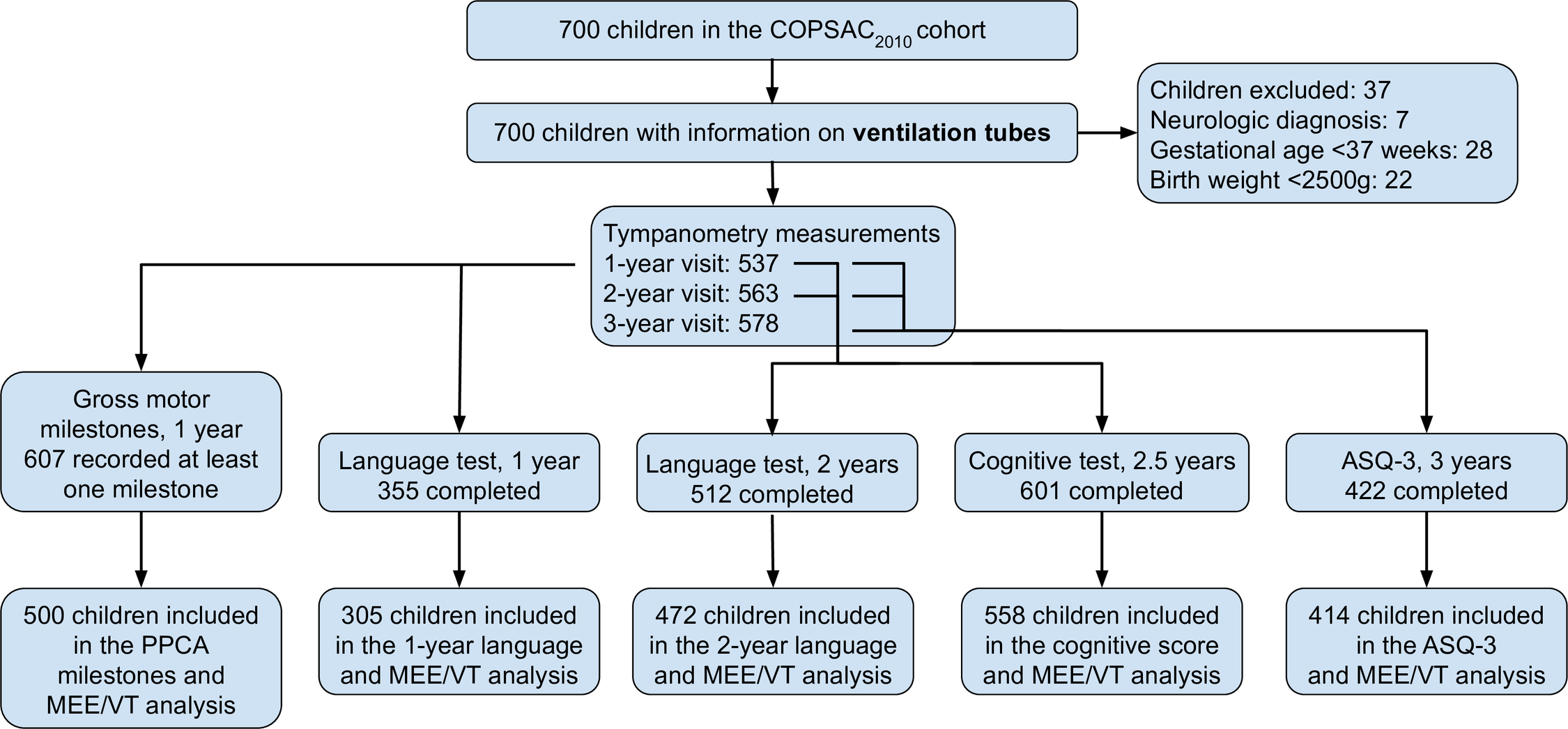

Supplement: S1 Fig — (TIF) [file pone.0280199.s001.tif]

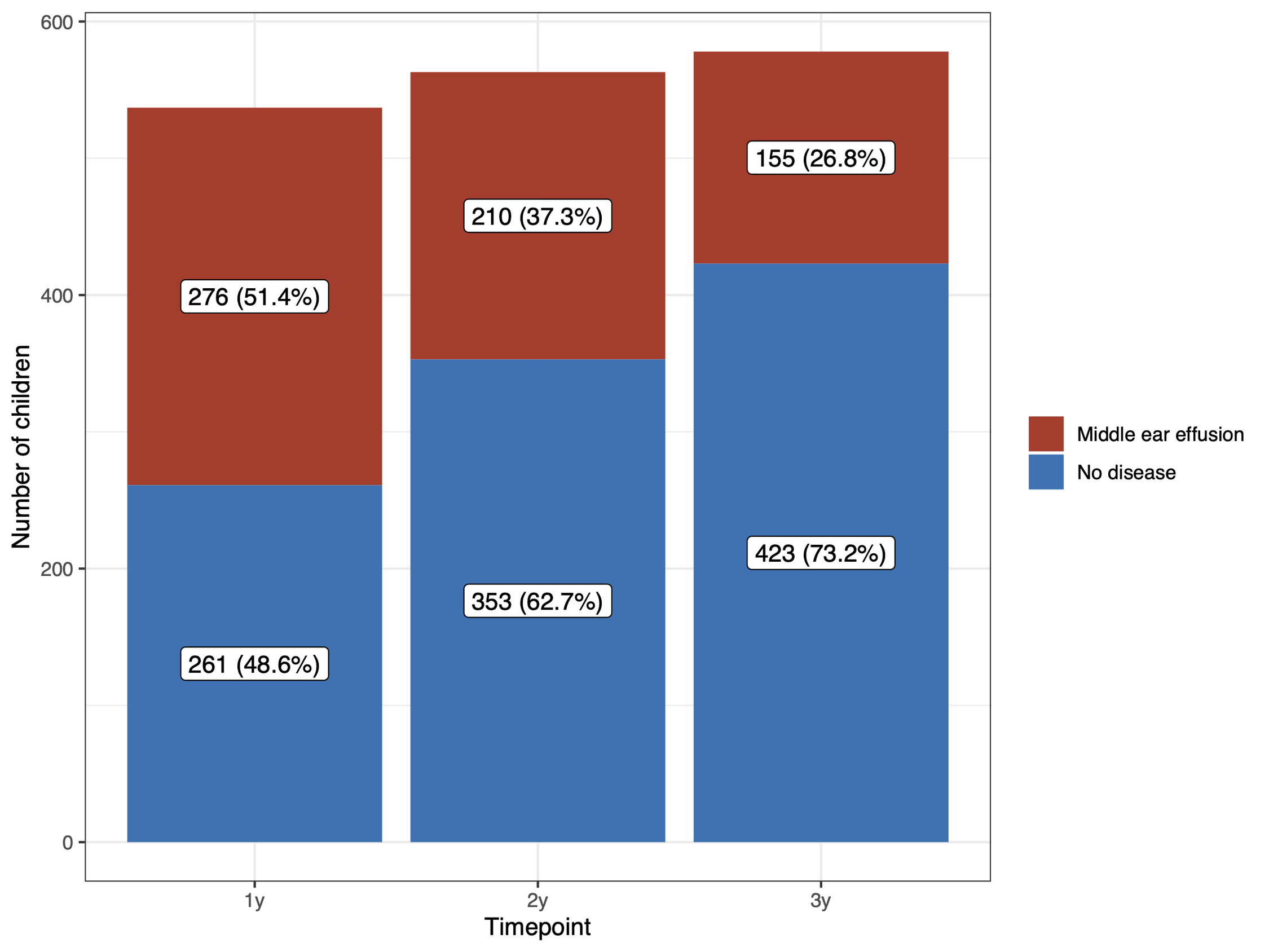

Supplement: S2 Fig — (TIF) [file pone.0280199.s002.tif]

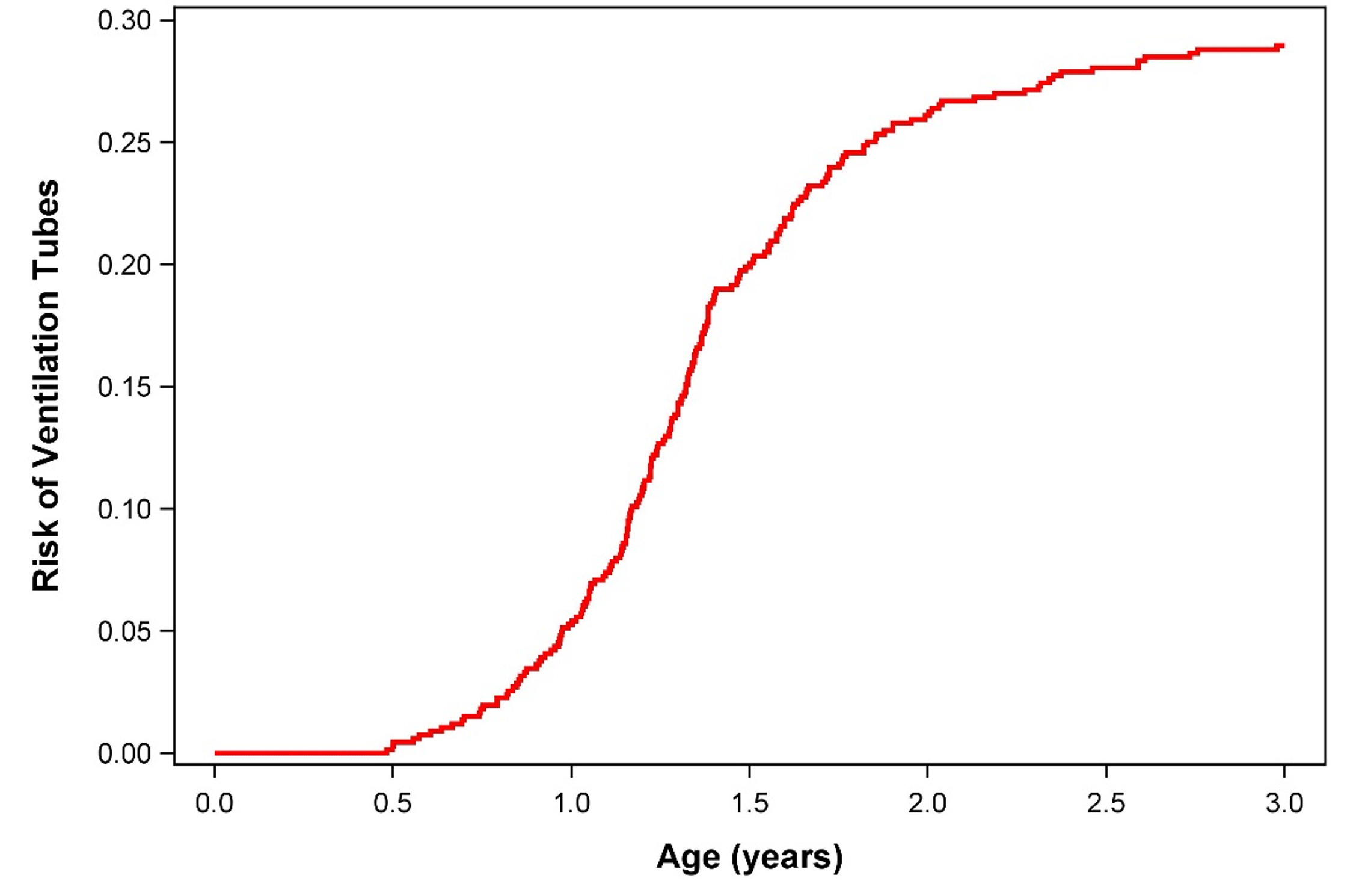

Supplement: S3 Fig — (TIF) [file pone.0280199.s003.tif]

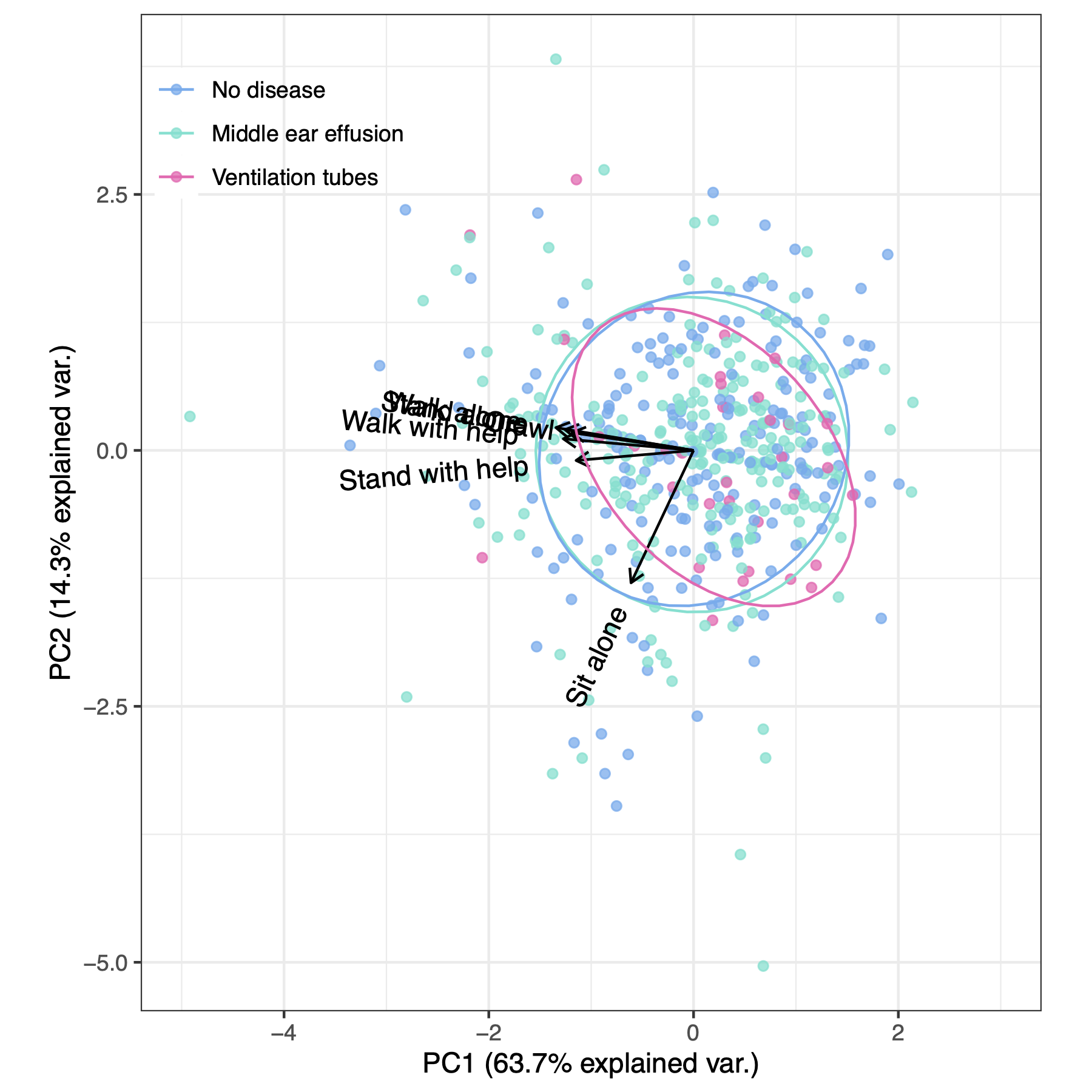

Supplement: S4 Fig — Ellipses illustrate the scores (95% CI) of children with middle ear effusion, treatment with ventilation tubes and no disease in the first year of life. The figure shows that there is no difference between children with middle ear effusion, ventilation tubes and no disease in the age of achieving the gross motor milestones. High PC1 scores can be interpreted as lower age at achievement of alle the gross motor milestones, especially Stand with help, Stand alone, Walk with help, Walk alone. (TIF) [file pone.0280199.s004.tif]

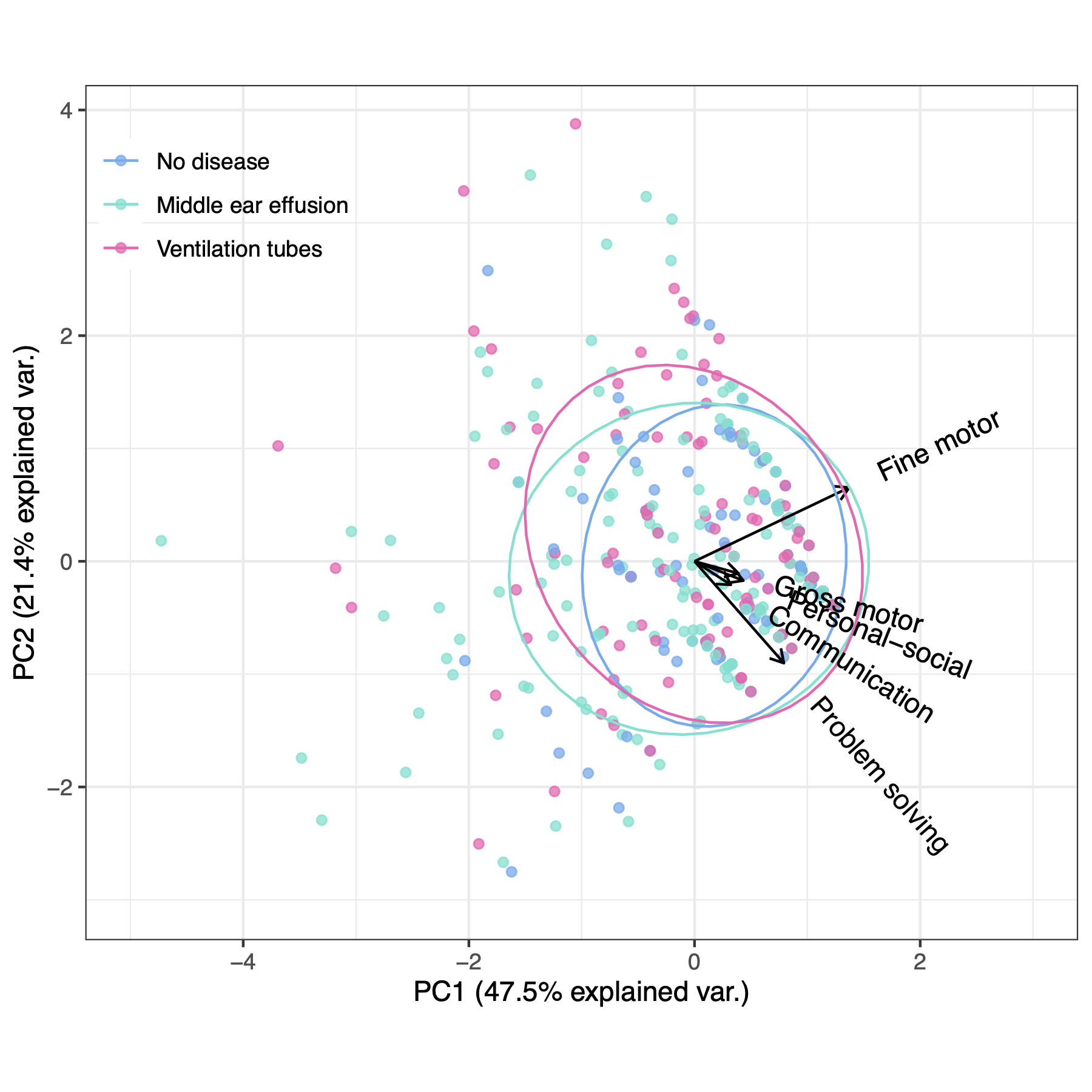

Supplement: S5 Fig — Ellipses illustrate the scores (95% CI) of children with middle ear effusion, treatment with ventilation tubes and children with no disease. The ASQ-3 consists of 5 categories; fine motor development, gross motor development, personal-social skills, communication and problem solving. When the scores are analyzed together as one measure of the child’s development it results in a PC1 score, which explains 47.5% of the variation. Higher values on PC1 equates to higher scores in all categories, especially Fine motor and Problem solving, which can be interpreted as children with high PC1 scores are further in their development than those with low PC1 scores. (TIF) [file pone.0280199.s005.tif]
