# Supplementary material for: Middle ear effusion, ventilation tubes and neurological development in childhood
Source: PLoS One. 2023 Jan 13;18(1):e0280199. doi: 10.1371/journal.pone.0280199 (PMC9838841; doi:10.1371/journal.pone.0280199)
Supplement: Attachment — Submitted filename: 221128_Response_to_Reviewers_R1.docx [file pone.0280199.s006.docx]

Dear Dr. Monsanto,

Thank you for the opportunity to resubmit our manuscript, which has now been updated and revised according to the points raised by you and the reviewers. Please find our responses below in blue.

Kind Regards

Jakob Stokholm

PONE-D-22-25558

Middle ear effusion, ventilation tubes and neurological development in childhood

PLOS ONE

Dear Dr. Stokholm,

Thank you for submitting your manuscript to PLOS ONE. After careful consideration, we feel that it has merit but does not fully meet PLOS ONE’s publication criteria as it currently stands. Therefore, we invite you to submit a revised version of the manuscript that addresses the points raised during the review process.

Although the study is interesting, there is a major concern regarding data availability. This is one of the requirements of Plos One (please see "Additional Editor Comments" section below). 

Please submit your revised manuscript by Jan 09 2023 11:59PM. If you will need more time than this to complete your revisions, please reply to this message or contact the journal office at [plosone@plos.org](mailto:plosone@plos.org). When you're ready to submit your revision, log on to <https://www.editorialmanager.com/pone/> and select the 'Submissions Needing Revision' folder to locate your manuscript file.

Please include the following items when submitting your revised manuscript:

- A rebuttal letter that responds to each point raised by the academic editor and reviewer(s). You should upload this letter as a separate file labeled 'Response to Reviewers'.
- A marked-up copy of your manuscript that highlights changes made to the original version. You should upload this as a separate file labeled 'Revised Manuscript with Track Changes'.
- An unmarked version of your revised paper without tracked changes. You should upload this as a separate file labeled 'Manuscript'.

If you would like to make changes to your financial disclosure, please include your updated statement in your cover letter. Guidelines for resubmitting your figure files are available below the reviewer comments at the end of this letter.

If applicable, we recommend that you deposit your laboratory protocols in [protocols.io](http://protocols.io) to enhance the reproducibility of your results. [Protocols.io](http://Protocols.io) assigns your protocol its own identifier (DOI) so that it can be cited independently in the future. For instructions see: [https://journals.plos.org/plosone/s/submission-guidelines#loc-laboratory-protocols](https://journals.plos.org/plosone/s/submission-guidelines#loc-laboratory-protocols). Additionally, PLOS ONE offers an option for publishing peer-reviewed Lab Protocol articles, which describe protocols hosted on [protocols.io](http://protocols.io). Read more information on sharing protocols at <https://plos.org/protocols?utm_medium=editorial-email&utm_source=authorletters&utm_campaign=protocols>.

We look forward to receiving your revised manuscript.

Kind regards,

Rafael da Costa Monsanto, M.D.
Academic Editor
PLOS ONE

Journal Requirements:

When submitting your revision, we need you to address these additional requirements.

1. Please ensure that your manuscript meets PLOS ONE's style requirements, including those for file naming. The PLOS ONE style templates can be found at

<https://journals.plos.org/plosone/s/file?id=wjVg/PLOSOne_formatting_sample_main_body.pdf> and

<https://journals.plos.org/plosone/s/file?id=ba62/PLOSOne_formatting_sample_title_authors_affiliations.pdf>

Reply: Done. The manuscript has been reformatted according to these specifications.

2. Thank you for stating the following in the Competing Interests section:

“I have read the journal's policy and the authors of this manuscript have the following competing interests: JT has received a speaking fee from AstraZeneca”

Please confirm that this does not alter your adherence to all PLOS ONE policies on sharing data and materials, by including the following statement: "This does not alter our adherence to  PLOS ONE policies on sharing data and materials.” (as detailed online in our guide for authors <http://journals.plos.org/plosone/s/competing-interests>).  If there are restrictions on sharing of data and/or materials, please state these. Please note that we cannot proceed with consideration of your article until this information has been declared.

Please include your updated Competing Interests statement in your cover letter; we will change the online submission form on your behalf.

Reply: Done. The updated competing interest section is included in both manuscript and cover letter.

3. In your Data Availability statement, you have not specified where the minimal data set underlying the results described in your manuscript can be found. PLOS defines a study's minimal data set as the underlying data used to reach the conclusions drawn in the manuscript and any additional data required to replicate the reported study findings in their entirety. All PLOS journals require that the minimal data set be made fully available. For more information about our data policy, please see <http://journals.plos.org/plosone/s/data-availability>.

Upon re-submitting your revised manuscript, please upload your study’s minimal underlying data set as either Supporting Information files or to a stable, public repository and include the relevant URLs, DOIs, or accession numbers within your revised cover letter. For a list of acceptable repositories, please see [http://journals.plos.org/plosone/s/data-availability#loc-recommended-repositories](http://journals.plos.org/plosone/s/data-availability#loc-recommended-repositories). Any potentially identifying patient information must be fully anonymized.

Important: If there are ethical or legal restrictions to sharing your data publicly, please explain these restrictions in detail. Please see our guidelines for more information on what we consider unacceptable restrictions to publicly sharing data: [http://journals.plos.org/plosone/s/data-availability#loc-unacceptable-data-access-restrictions](http://journals.plos.org/plosone/s/data-availability#loc-unacceptable-data-access-restrictions). Note that it is not acceptable for the authors to be the sole named individuals responsible for ensuring data access.

We will update your Data Availability statement to reflect the information you provide in your cover letter.

Reply: Done. The updated data availability section is now included in the manuscript file and copied into the cover letter.

4. Please include captions for your Supporting Information files at the end of your manuscript, and update any in-text citations to match accordingly. Please see our Supporting Information guidelines for more information: <http://journals.plos.org/plosone/s/supporting-information>.

Reply: Done. The supporting information files are now uploaded separately, and the captions are included at the end of the manuscript file.

Additional Editor Comments:

One major concern the editorial board has is regarding the following statement:

"We note your Data Availability statement: "Individual-level clinical data from the children participating in the cohort cannot be made freely available, to protect the privacy of the participants and their families. However, research collaborations are welcome and data can be made available under a joint research collaboration by contacting the corresponding author."

PLOS journals require authors to make all data necessary to replicate their study’s findings publicly available without restriction at the time of publication. When specific legal or ethical restrictions prohibit public sharing of a data set, authors must indicate how others may obtain access to the data.

Please address the following:

a) At this time disclose the reason for this data restriction and provide a point of contact, preferably an email, for the ethical body mandating the restriction of this data. Please note that this point of contact cannot be an author of this study.

b) If there are no restrictions on this dataset, please provide your data by uploading it as a Supporting Information file or depositing it in a stable repository.

We hope to hear from you soon.

Reply: Thank you for highlighting this. We have amended the manuscript to further specify the reasoning for restricted access to data and have included an email where requests for data access can be sent: COPSAC Data Protection Officer (DPO) Ulrik Ralfkiaer, PhD (administration@dbac.dk).

[Note: HTML markup is below. Please do not edit.]

Reviewers' comments:

Reviewer's Responses to Questions

**Comments to the Author**

1. Is the manuscript technically sound, and do the data support the conclusions?

The manuscript must describe a technically sound piece of scientific research with data that supports the conclusions. Experiments must have been conducted rigorously, with appropriate controls, replication, and sample sizes. The conclusions must be drawn appropriately based on the data presented.

Reviewer #1: Yes

Reviewer #2: Partly

2. Has the statistical analysis been performed appropriately and rigorously?

Reviewer #1: Yes

Reviewer #2: N/A

3. Have the authors made all data underlying the findings in their manuscript fully available?

The [PLOS Data policy](http://www.plosone.org/static/policies.action#sharing) requires authors to make all data underlying the findings described in their manuscript fully available without restriction, with rare exception (please refer to the Data Availability Statement in the manuscript PDF file). The data should be provided as part of the manuscript or its supporting information, or deposited to a public repository. For example, in addition to summary statistics, the data points behind means, medians and variance measures should be available. If there are restrictions on publicly sharing data—e.g. participant privacy or use of data from a third party—those must be specified.

Reviewer #1: Yes

Reviewer #2: Yes

4. Is the manuscript presented in an intelligible fashion and written in standard English?

PLOS ONE does not copyedit accepted manuscripts, so the language in submitted articles must be clear, correct, and unambiguous. Any typographical or grammatical errors should be corrected at revision, so please note any specific errors here.

Reviewer #1: Yes

Reviewer #2: Yes

5. Review Comments to the Author

Please use the space provided to explain your answers to the questions above. You may also include additional comments for the author, including concerns about dual publication, research ethics, or publication ethics. (Please upload your review as an attachment if it exceeds 20,000 characters)

Reviewer #1: The issue of the effects of acute otitis media on motor and language development still needs to be addressed. Therefore, cohorts with long periods are studies that can help to understand the real causal involvement of the inflammatory processes of the middle ear and the treatment with the placement of the ventilation tube.

Comment 1 (C1): I congratulate authors for this manuscript. It is very well written, with a huge sample providing relevant data due to its 3-year follow-up, apparently with little missing data for different endpoint analyzed.

Reply 1 (R1): Thank you for these accolades.

C2: However, I would like to point out one aspect that need clarification.
- In the results in relation to the Baseline Characteristics, it was confusing because it is very clear that of the 700 children followed, only 37 were excluded in relation to the placement of Ventilation tubes, however, when reporting the % of ESM diagnosis with tympanometry, it is reported that 51% (n= 276/537) had MEE at 1 year, 37% (n=210/563) at 2 years and 26% (n=155/578) at 3 years. Are these numbers 537, 563, 578 for the different years and not 663 due to missing data? Please make this clearer in the text.

R2: Thank you for bringing this to our attention. Indeed, of the 663 children included in the study, 537 had tympanometry performed at 1 year, 563 at 2 years and 578 at 3 years. We have now clarified this in the results section, where the pertinent section now looks like this:

*“A total of 700 children were included in the COPSAC_2010_ cohort at birth and information on VT insertions was available for all 700 children. We excluded 37 children from the study because of neurological diagnosis, low gestational age, or low birth weight (S1 Fig), leaving 663 children. In these 663 children, tympanometry was successfully performed on 537 children at 1 year, 563 at 2 years, and 578 at 3 years (S1 Fig), showing that 51% (n=276/537) had MEE at 1 year, 37% (n=210/563) at 2 years and 26% (n=155/578) at 3 years (S2 Fig).”*

Reviewer #2: Dear authors:

Although the manuscript is generally interesting, I have a few concerns:


C3: ABSTRACT
Should be adjusted as per the suggestions below.
R3: Thanks, we have now updated the abstract introduction and conclusion.

C4: INTRODUCTION
Although your manuscript is generally interesting, it was unclear how your work adds to the already existing literature in this regard. The introduction is highly summarized and does not address the literature gap that the study aims to fill. I would strongly recommend that the authors reformulate their introduction to address this.
R4: We have now amended the introduction to highlight how our contribution can add to the field.

METHODS
The exclusion criteria should be better delineated:
C5: In ‘Study Population’ the phrase: - “The key exclusion criteria were chronic disease other than asthma or lack of fluency in Danish”. Does it refer to the mother or the child?
R5: This refers to the mother, which has now been clarified in the text.

C6: In ‘Middle ear effusion and ventilation tubes’ the phases: - “while type A or C tympanograms were considered normal” and “c) No disease (type A/C curve)”, should be better described, as a type C tympanogram, especially in a child, should never be considered normal.
R6: Thank you for highlighting this, we agree that the wording here was misleading. What was intended is to differentiate MEE from not MEE. Thus, we have now made the wording more precise in these sections:

*“A type B tympanogram (flat curve) was interpreted as MEE[18,19], while type A or C tympanograms were considered not MEE”*

and

*“For the outcome analyses, we defined a compound MEE/VT variable at each yearly timepoint as either a) Ventilation tube insertion before that date, b) Middle ear effusion (type B curve) at visit or at previous visit(s), c) No VT/MEE (type A/C curve) at visit, d) Missing data (no tympanometry measurement) at visit, in prioritized order.”*

C7: In ‘Language development’ it is described that: “Bilingual children were excluded from this assessment”. It is important to explain why and also put in the exclusion criteria.
R7: We have now updated this description with a justification:

*“Bilingual children were excluded from this assessment, since they may follow different temporal language acquisition patterns[27].”*

Since it is not an exclusion criterion from the study, but only from the language assessments, we think it is most appropriate to describe in this section.

C8: In ‘Covariates’ the authors cite that they excluded “37 children from the study because of neurological diagnosis, low gestational age, or low birth weight”. it should be in the exclusion criteria
R8: This is already stated in the Materials and Methods, under Study Population (*“For the present study, we excluded children with a neurological diagnosis and those born <37 weeks of gestation or with birth weight <2500g from all analyses.”*). Please note that we here distinguish between excluded from the cohort (n=700) and for this particular study (n=663).

C9: I believe that the data on the hearing thresholds of these children would be very enriching, as the degree of hearing loss greatly influences language acquisition.
R9: We agree with this comment – however, we unfortunately do not have any data on hearing thresholds for this cohort.

RESULTS
C10: It would be important to evaluate information regarding: “Gross motor milestones and Cognitive score”, because the data are accounted for with 700 children, but in the results, it was mentioned that some were excluded.
R10: Thanks for highlighting this, we agree it ought to be presented out of the 663 children in these sections. We have updated denominators and percentages throughout, and also updated intermediate numbers in fig S1 accordingly.

C11: Tables: Tables are confusing and are not consistent with usual scientific standards.
R11: We assume that the reviewer is referring primarily to table 2, since table 1 is a commonly used format in cohort research (baseline characteristics table). We can appreciate that table 2 could be confusing at first, since it is presenting a lot of very different neurological outcomes. Notably, the outcomes are measured as either count data (word production/comprehension) or continuous (principal components, cognitive and development scores). To add to that, the scales of these outcomes are all different. However, we chose to structure the table with the same type of exposure definition (MEE, VT, vs reference level of no disease) across all the outcomes to enable the reader to easily assess directionality, and with a bit more effort, reference text, figures, and supplemental figures to assess the scale of each outcome and thus the magnitude of the associations presented. Further suggestions for how the presentation can be clarified would be welcomed.

C12: DISCUSSION
Discussion is largely based on a repetition of the results. I missed a more in-depth critical analysis of their results, especially on how your results compares with those of previous studies.

R12: We have now expanded the section and referenced more studies in a more direct manner, while reducing the parts of the interpretation that summarized our results before relevant comparisons to other studies. Notably, we take care not to overinterpret our results in light of our study’s limitations (observational design, tympanometry as only clinical test, no hearing assessments).
